# Solar Simulated Ultraviolet Radiation Induces Global Histone Hypoacetylation in Human Keratinocytes

**DOI:** 10.1371/journal.pone.0150175

**Published:** 2016-02-26

**Authors:** Xiaoru Zhang, Thomas Kluz, Lisa Gesumaria, Mary S. Matsui, Max Costa, Hong Sun

**Affiliations:** 1 New York University, Department of Environmental Medicine, Tuxedo, New York, United States of America; 2 Estee Lauder Companies, Inc., Melville, New York, United States of America; Columbia University Medical Center, UNITED STATES

## Abstract

Ultraviolet radiation (UVR) from sunlight is the primary effector of skin DNA damage. Chromatin remodeling and histone post-translational modification (PTM) are critical factors in repairing DNA damage and maintaining genomic integrity, however, the dynamic changes of histone marks in response to solar UVR are not well characterized. Here we report global changes in histone PTMs induced by solar simulated UVR (ssUVR). A decrease in lysine acetylation of histones H3 and H4, particularly at positions of H3 lysine 9, lysine 56, H4 lysine 5, and lysine 16, was found in human keratinocytes exposed to ssUVR. These acetylation changes were highly associated with ssUVR in a dose-dependent and time-specific manner. Interestingly, H4K16ac, a mark that is crucial for higher order chromatin structure, exhibited a persistent reduction by ssUVR that was transmitted through multiple cell divisions. In addition, the enzymatic activities of histone acetyltransferases were significantly reduced in irradiated cells, which may account for decreased global acetylation. Moreover, depletion of histone deacetylase SIRT1 in keratinocytes rescued ssUVR-induced H4K16 hypoacetylation. These results indicate that ssUVR affects both HDAC and HAT activities, leading to reduced histone acetylation.

## Introduction

Solar ultraviolet radiation (UVR) leads to an array of photodamage endpoints, including sunburn, erythema and edema, as well as skin cancers [1]. Epidemiological studies have established an association between sunlight exposure and increased risk of melanoma and non-melanoma skin cancers. Solar radiation is composed of wavelengths in the UVA (320–400 nm), UVB (290–320 nm) and UVC (200–290 nm) ranges, however, due to the atmospheric ozone layer that blocks UVC and most of UVB, the UVR reaching the earth’s surface is a mixture of UVA (90–95%) and UVB (5–10%).

Both UVB and UVA are able to induce DNA damage in mammalian cells. UVB is absorbed by DNA and induces two major types of DNA lesions: cyclobutane pyrimidine dimers (CPDs) and (6–4) pyrimidine-pyrimidone photoproducts [(6–4)PPs]. In contrast, UVA induces reactive oxygen species (ROS) that generate oxidative DNA damage (such as 8-oxo-deoxyguanine) [2, 3]. Failure to repair these DNA lesions can result in either cell death or accumulation of DNA mutations, which in turn may lead to skin tumor formation and progression. Thus, DNA damage/mutation and oxidative stress have been considered as key initiating and promoting events underlying ssUVR-induced skin cancers.

Mammalian cells have developed a highly sophisticated defense response (DNA damage response, DDR) to protect their genomic integrity. Multiple DNA repair pathways, including nucleotide excision repair (NER), base excision repair (BER), homologous recombination repair (HRR) and non-homologous end-joining (NHEJ), are actively involved in repairing UVR-induced DNA damage [2, 3]. Simultaneously, DNA replication and transcription are temporarily stalled at the damaged sites; chromosome segregation is paused at cell cycle checkpoints. Successful DNA repair requires coordination between these chromatin-associated events, as well as functional chromatin remodeling machinery that precisely controls DNA accessibility [4].

In addition to chromatin remodeling complexes, histones and their post-translational modifications (PTMs) play an important role in chromatin structural changes [4]. More than 8 histone modifications have been identified, including acetylation, methylation, phosphorylation, ubiquitination, sumoylation, ADP ribosylation, deamination and biotinylation, etc. These PTMs exhibit either site-specific or modification-specific effects in almost all chromatin-related cellular processes, such as DNA replication, transcription, cell cycle progression, as well as every step of the DNA damage response [5]. For example, phosphorylation of histone H2A variant (γ-H2AX), methylation of histone H3 lysine 79, and histone acetylation on histone H3 lysine 9 and lysine 14 have been connected to damage sensing and chromatin opening, while dephosphorylation of H2AX, acetylation or deacetylation of histone H3 and H4 were found to be important to chromatin restoration [6–8].

It is worth noting that most of the information on histone PTMs and DDR has been obtained from studies on DNA double strand breaks (DSB) induced by ionizing radiation or a well defined UVB or UVC radiation. Very little is known about histone PTM changes in mammalian skin cells in response to solar UVR, a mixture of UVA and UVB. In the present study, we analyzed the changes of histone PTMs in human keratinocytes exposed to solar simulated UVR (ssUVR). Global acetylation of several lysine residues located on histone H3 and H4 were reduced after either a single dose of radiation or 3–5 repetitive exposures of ssUVR. Interestingly, the activities of enzymes that deposit or remove acetyl groups were also affected, which may account for the hypoacetylation of histone H3 and H4 in ssUVR irradiated cells.

## Materials and Methods

### Cell culture

Immortalized human HaCaT keratinocytes, a kind gift from Dr. Chuangshu Huang at NYU School of Medicine [9], were cultured in DMEM medium (4.5 g/L glucose) containing 10% fetal bovine serum and 1% penicillin—streptomycin at 37°C and 5% CO_2_. Normal human epidermal keratinocytes (NHEK) were obtained from Lonza (Walkersville, MD), and maintained in KGM-Gold^™^ keratinocyte growth medium (Lonza, Walkersville, MD).

### UV source and simulated UV radiation

Solar simulated UV radiation was performed using a modified Hand Foot II phototherapy instrument (National Biological Corporation, Beachwood, OH) with 8 Houvalite UVA lamps. The lamp emission is filtered by a single glass plate, resulting in a spectrum that contains 95% UVA and 5% UVB. The intensity of the lamp output was measured before every exposure using an UVX Radiometer with UVA and the exposure times were calculated to deliver the desired doses. UVB intensity was measured with an ILT1400 Photo detector using the SEL240 UVB sensor (International Light, Peabody, MA). The range of doses used in the study is comparable to what a person would be exposed to when standing outside for 40 minutes (3 J/cm^2^) to 4 hours (18 J/cm^2^) at noon under a clear sky with a UV index of 6 or higher [10].

Cells were seeded in 10-cm cell culture dish at a density of 3.5 x 10^6^ cells per dish. Prior to exposure, cells were washed twice with PBS and exposed to ssUVR in 10 ml of Hank’s Balanced Salt Solution (HBSS, life technologies, Grand Island, NY) supplemented with 4 mM glucose for the required exposure time specified. The solar simulated UV radiation used in this study is much less intense compared to the UVB and UVC alone. It takes a longer time interval to achieve an appropriate dosage. Therefore, cells in 10-cm dish were incubated with 10 ml HBSS during irradiation to prevent desiccation. After irradiation, the HBSS was replaced with fresh medium and cells were cultured for 24 hours. Control samples were treated identically but covered with foil during irradiation (Sham). To maintain a constant temperature, a table fan was used to reduce heat production during the exposure. For repetitive radiation, cells were exposed as described above, except they were allowed to recover for 3 days prior to the next round of radiation.

### γH2AX staining and immunofluorescence microscopy

Cells were seed in 6-cm cell culture dishes. On the Next day, cells were exposed to various doses of ssUVR (0, 3, 12, 18 J/cm^2^). At 1 hour after irradiation, cells were fixed with 100% cold methanol at -20°C for 15 minutes, and permeabilized with 0.2% Triton X-100 for 10 minutes. The cells were then blocked with 3% BSA in PBS for 1 hour at room temperature, and incubated overnight at 4°C with γH2AX monoclonal antibody (Abcam, ab26350). After three washes, cells were incubated with the secondary antibody conjugated with Alexa Fluor 488 (Molecular Probes) for 1 hour at room temperature. The cells were mounted with Prolong Diamond Antifade reagent containing DAPI (Molecular Probes). Images were acquired using Leica SP5 Confocal microscope. The intensity of γH2AX signals was quantified using ImageJ. More than 50 cells were analyzed in each sample, and the results were presented as the relative intensity compared to sham.

### Cell viability assay

Cells were seeded in 6-cm cell culture dishes the evening before irradiation. Cells were exposed to various doses of ssUVR (0, 1, 6, 12, and 18 J/cm^2^). Irradiated cells were harvested at 24 hours after exposure. Cell viability was assessed using Trypan Blue exclusion. Both viable and nonviable cells were counted, and cell viability was presented as the percentage of viable cells relative to the total cell numbers. Two independent experiments were performed, each with duplicate samples.

### Whole cell lysates and Western blot analysis

For collecting whole cell lysate, cells were lysed with boiling buffer (1% SDS, 10 mM Tris (pH 7.4), 1 mM sodium orthovanadate). 50 μg of whole cell lysate were separated by 15% SDS-PAGE and transferred to the nitrocellulose membrane (GE Life Science, Piscataway, NJ). Membranes were blocked and incubated with primary and appropriate secondary antibodies. The bound antibodies were detected and visualized using chemiluminescence reagents. Two or three independent experiments were performed for each data set. Histone H3 was used to access protein loading for each lane. For western blot quantification, the intensity of the bands was quantified using ImageJ Software. The intensity of each band was normalized to that of histone H3.

The antibodies for H3K4me3 (ab8580), H3K9me2 (ab1220), H3K27me3 (ab6002), H3K9ac (ab4441), H3K14ac (ab52946), H3K18ac (ab1191), H3K56ac (ab76307), H4K5ac (ab51997), H4K12ac (ab61238), H4K16ac (ab61240), were purchased from Abcam. Antibodies for Pan H3ac (06–599), H4K16ac (07–329) are from Millipore. Anti-Sirt1 antibody (2496) is from Cell Signaling.

### Nuclear extracts

Nuclear fraction from sham and ssUV irradiated cells were isolated using nuclear extraction kit (Active Motif, Carlsbad, CA) according to the manufacture’s instruction. The concentrations of nuclear extracts were determined by Dc protein assay (Bio-Rad, Hercules, CA) using bovine serum albumin as the standard.

### HAT and HDAC activities

20 μg of nuclear extracts from sham and ssUV radiated cells were used to analyze the enzyme activities of histone acetylatransferase (HAT) and deacetylase (HDAC). HAT activity was analyzed by HAT activity assay kit (Active Motif, Carlsbad, CA) using either H3 or H4 peptides as the substrates. The nuclear extracts were incubated with either histone H3 or H4 peptides in a mixture containing acetyl-CoA for 1 hour at room temperature. After the reaction was terminated with stop solution, the samples were incubated with developing solution for additional 15 minutes. The fluorescence in each sample was assessed with excitation wavelength at 360 nm and emission wavelength at 460 nm using a Spectra Max Gemini (Molecular Devices). HDAC activity was measured using the fluorescent HDAC assay kit (Active motif, Cartsbad, CA). For specify the different classes of HDAC, Trichostatin A (TSA) (Sigma, St. Louis, MO) were used to inhibit class I/II HDACs. Briefly, the nuclear extracts were incubated with HDAC substrate in 96-well plate at 37°C for 60 minutes, and followed by incubation with HDAC developer solution for additional 15 minutes. The fluorescence was assessed at an excitation wavelength of 365 and emission wavelength of 460 nm.

### Small RNA interference

The On-TARGETplus SMARTpool siRNAs against human Sirt1 (Dharmacon) were used to transiently knockdown Sirt1 in HaCaT cells. Briefly, HaCaT cells were transfected with Sirt1 siRNA or scramble siRNA using Lipofectamine RNAiMax (Invitrogen). 48 hours after transfection, the cells were split, and subsequently irradiated with ssUV at 12 J/cm^2^ at next day. The irradiated cells were collected 24 hours later, and analyzed for Sirt1 protein levels and histone acetylation.

### Statistical Analysis

For analysis of statistical significance, the Student's *t*-test was used for all calculations, and P-values of <0.05 were considered to be statistically significant.

## Results

### ssUV radiation modulates histone post transcriptional marks

**Fig 1:**
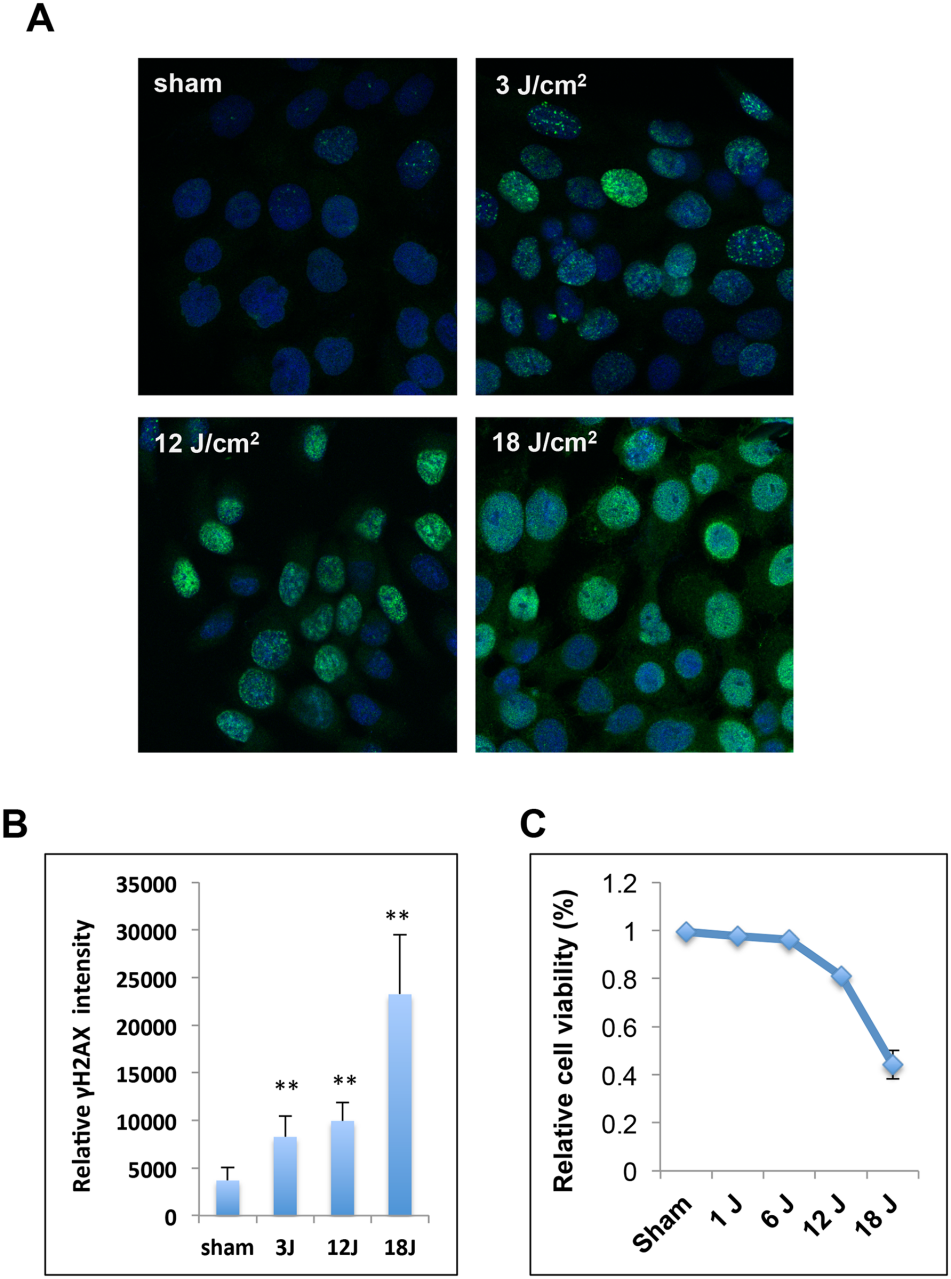
H2AX phosphorylation and cell viability after ssUV irradiation. (A, B) ssUVR induces γH2AX in human keratinocytes. HaCaT cells were exposed to ssUVR at 0, 3, 12, 18 J/cm^2^, the levels of γH2AX was determined at 1 hour after irradiation. (A) Representative image. (B) Relative γH2AX intensity in irradiated HaCaT cells. γH2AX intensity was quantified as the corrected total cell fluorescence and presented as the mean ± SD (n = 54). Statistical significance of the difference between sham and ssUVR-treated cells was analyzed using Student's *t*-test. ** p<0.01 (C) HaCaT cells were exposed to various doses of ssUVR (0, 1, 6, 12, and 18 J/cm^2^). Cell viability was assessed at 24 hours after exposure, and presented as the percentage of viable cells relative to the total cell numbers. The results showed the mean ± SD (n = 4)

Phosphorylation of histone variant H2AX at Ser-139 (γH2AX) is one of the best-characterized histone PTM involved in DNA damage response. It has been reported that UV radiation induced γH2AX in many different type of cells [11, 12]. The effects of ssUVR on H2AX phosphorylation and cell viability were analyzed to select the proper dose range of ssUVR used in this study. Exponentially growing human keratinocyte HaCaT cells were exposed to ssUVR at 0, 3, 12, 18 J/cm^2^, the levels of γH2AX was determined at 1 hour after irradiation using immunofluorescence microscopy. As shown in Fig 1A, while there are a few cells exhibited dim nuclear γH2AX foci in unirradiated control sample (sham), ssUV irradiated cells exhibited a signi ficant increase of γH2AX signals (Fig 1B). Cells exposed to 3 or 12 J/cm^2^ displayed about 2-fold increase of γH2AX signals within 1 hour, however, cells exposed to 18 J/cm^2^ exhibited a robust increased γH2AX levels (~ 5-fold) (Fig 1B). Interestingly, similar dose response was observed in cell viability assay. As cells exposed to 12 J/cm^2^ retained about 80% viability after 24 hours, less then half of cells exposed to 18 J/cm^2^ are viable (Fig 1C). Thus, 12 J/cm^2^ was used in most of studies presented in this paper.

To test whether ssUV radiation can alter the posttranscriptional marks located in histone tails, the HaCaT cells were exposed to either a single ssUV radiation or three repetitive radiations (once every three days) at 12 J/cm^2^. Cells were harvested at 24 hours after the last radiation, and analyzed for the histone marks by western blot using specific antibodies. Giving the established role of histone methylation and acetylation in gene expression and DDR, we initiated our study by analyzing several methylation and acetylation marks.

**Fig 2:**
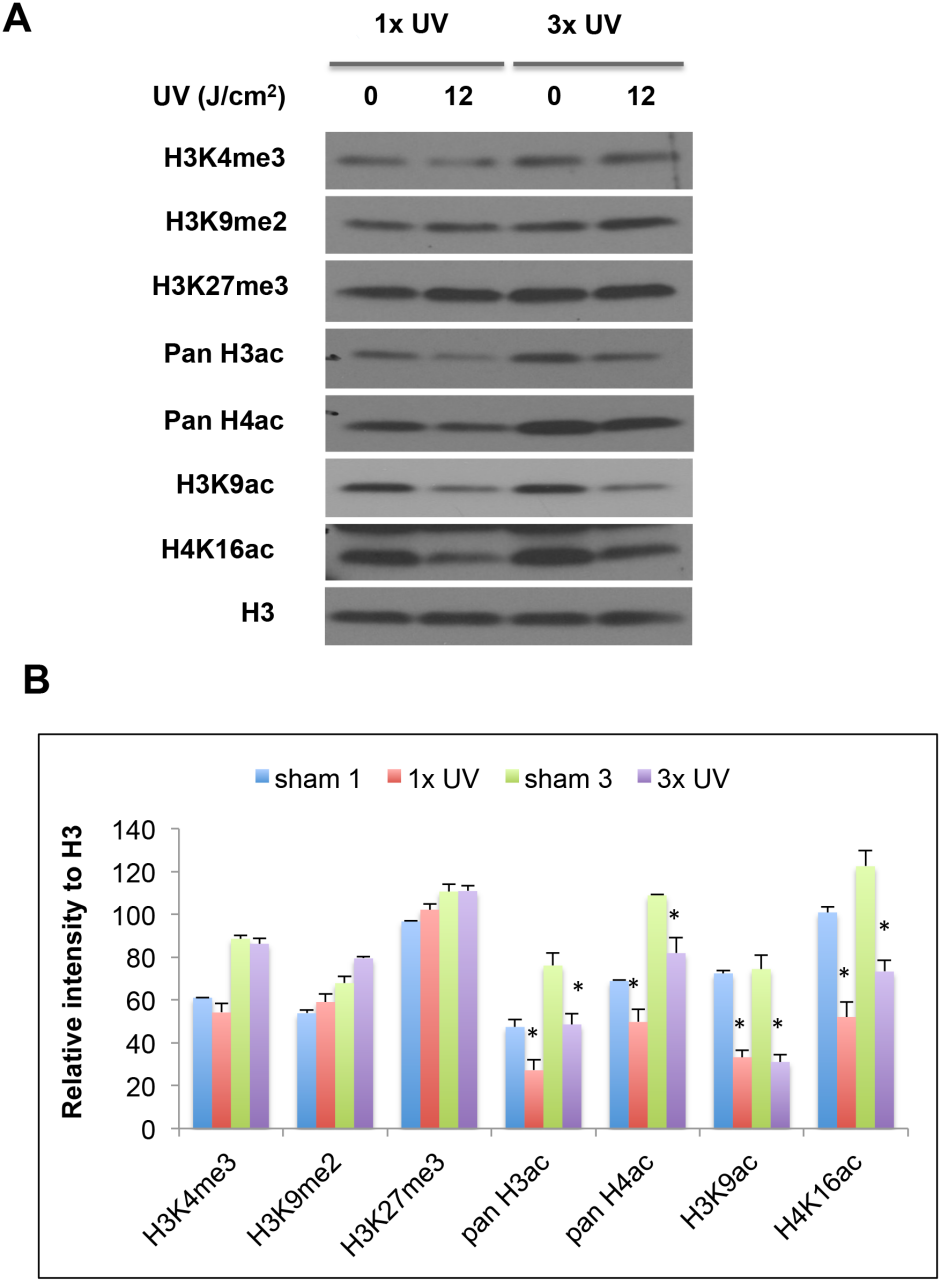
Modulation of histone PTMs by ssUVR. HaCaT cells were exposed to a single dose of ssUV (1xUV) at 12 J/cm^2^ or 3 repetitive radiations of the same dose (3xUV). Cells were harvested at 24 hr after the last exposure, and the levels of H3K4me3, H3K9me2, H3K27me3, pan H3ac, pan H4ac, H3K9ac, and H4K16ac were analyzed by western blot analysis using specific antibodies. An antibody against histone H3 was used to assess the loading of samples in each lane. (A) Representative western blot. (B) The relative intensity of the bands was presented as the ratio of each band versus its corresponding H3. The results from replicate experiments were plotted as the mean ± SD (n = 3). Statistical significance of the difference between sham and ssUVR-treated cells was analyzed using Student's *t*-test. * *P* < 0.05.

Three different methylation marks, tri-methylated H3 lysine 4 (H3K4me3), di-methylated histone H3 lysine 9 (H3K9me2) and tri-methylated histone H3 lysine 27 (H3K27me3), were selected based on their roles in controlling gene expression and cellular stress response [13–16]. However, none of these marks was significantly altered at the global levels in HaCaT cells exposed to either a single dose or 3 repetitive ssUVR (Fig 2).

Histone acetylation is normally associated with active gene expression and is crucial for DNA damage response [5, 17, 18]. Antibodies that recognize the acetylation of multiple lysine residues on either histone H3 or H4 were used to test the effect of ssUVR on histone acetylation. Pan H3ac antibody recognizes the acetyl group in both lysine 9 and lysine 14, and pan H4ac antibody reacts with all four acetylated lysine residues located in the N terminal of H4, including lysine 5, lysine 8, lysine 12 and lysine 16. As shown in Fig 2, both pan H3 acetylation and pan H4 acetylation were reduced in ssUV-irradiated cells compared to Sham. We also used antibodies specifically against histone acetylation at H3 lysine 9 and H4 lysine 16. Both acetylation marks were reduced as well (Fig 2). This reduction of histone acetylation can be seen in both single and repetitive exposure, suggesting a strong link between ssUVR and histone acetylation changes.

### ssUVR induces hypoacetylation in histone H3 and H4

**Fig 3:**
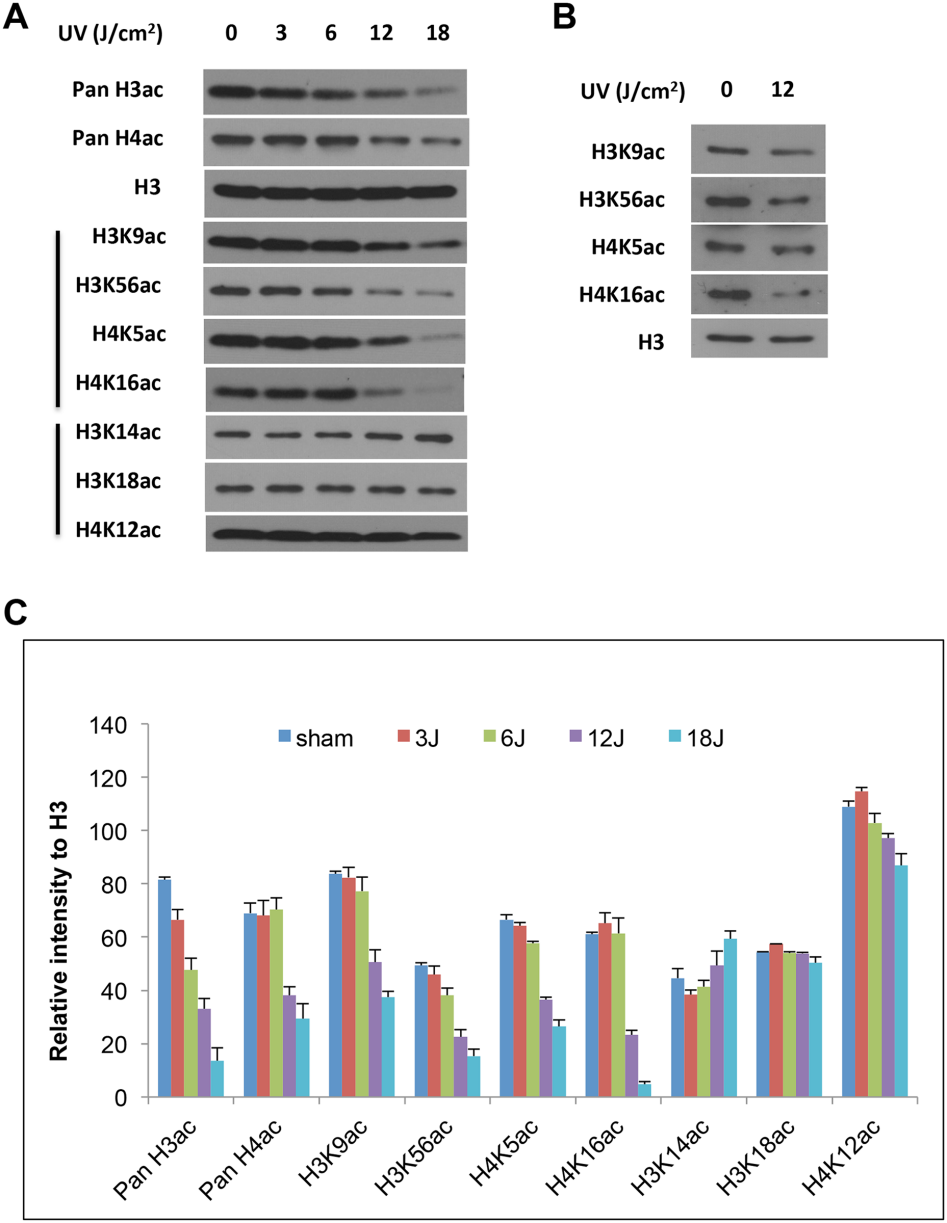
ssUVR induces reductions in histone acetylation in a dose-dependent manner. (**A**) Dose-dependent decreases of histone acetylation by ssUVR. HaCaT cells were exposed to a single ssUVR at 3, 6, 12, and 18 J/cm^2^, and cells were harvested 24 hr after irradiation. (B) The relative intensity of the bands was presented as the ratio of each band versus its corresponding H3. The results from replicate experiments were plotted as the mean ± SD (n = 3). (**C**) NHEK cells were exposed to a single ssUVR at 12 J/cm^2^, and cells were harvested at 24 hours after irradiation. The levels of several histone acetylation marks on histone H3 and H4 were analyzed by western blot analysis using specific antibodies. An antibody against histone H3 was used to assess the loading of samples in each lane.

Subsequent experiments focused on histone acetylation since methylation changes were not observed following ssUVR. HaCaT cells were exposed to various doses of ssUVR (3, 6, 12, and 18 J/cm^2^). Irradiated cells were harvested at 24 hours after exposure, and subjected to analysis of histone acetylation. As shown in Fig 3A, the levels of pan H3ac were reduced by ssUVR in a dose-dependent manner. To determine which mark was affected by ssUVR, we used antibodies specifically against each individual PTM in subsequent studies. In addition, acetylation of histone H3 lysine 18 and lysine 56 has recently been implicated in DDR were also examined [18–20]. The change in histone acetylation marks can be categorized into two distinct groups based on their response to UVR. While three marks (H3K14ac, H3K18ac, and H4K12ac) were seemingly unaffected, the other four marks, including H3K9ac, H3K56ac, H4K5ac and H4K16ac, exhibited a remarkable reduction in HaCaT cells exposed to ssUVR (Fig 3A and 3B). Similar to pan H3ac and H4ac, decreased histone acetylation in these four sites was more profound in cells exposed to higher doses of ssUVR (12 and 18 J/ cm^2^). These results suggested a global hypoacetylation of histone as an outcome of acute ssUV radiation.

To investigate whether ssUVR induces histone hypoacetylation in other cell types, normal human epidermal keratinocytes (NHEKs) were exposed to a single 12 J/cm^2^ ssUVR. NHEK cells were harvested at 24 hours after irradiation, and were analyzed for histone acetylation. As shown in Fig 3C, acetylation of histone H3K9, H3K56, H4K5 and H4K16 were reduced in cells exposed to ssUVR.

**Fig 4:**
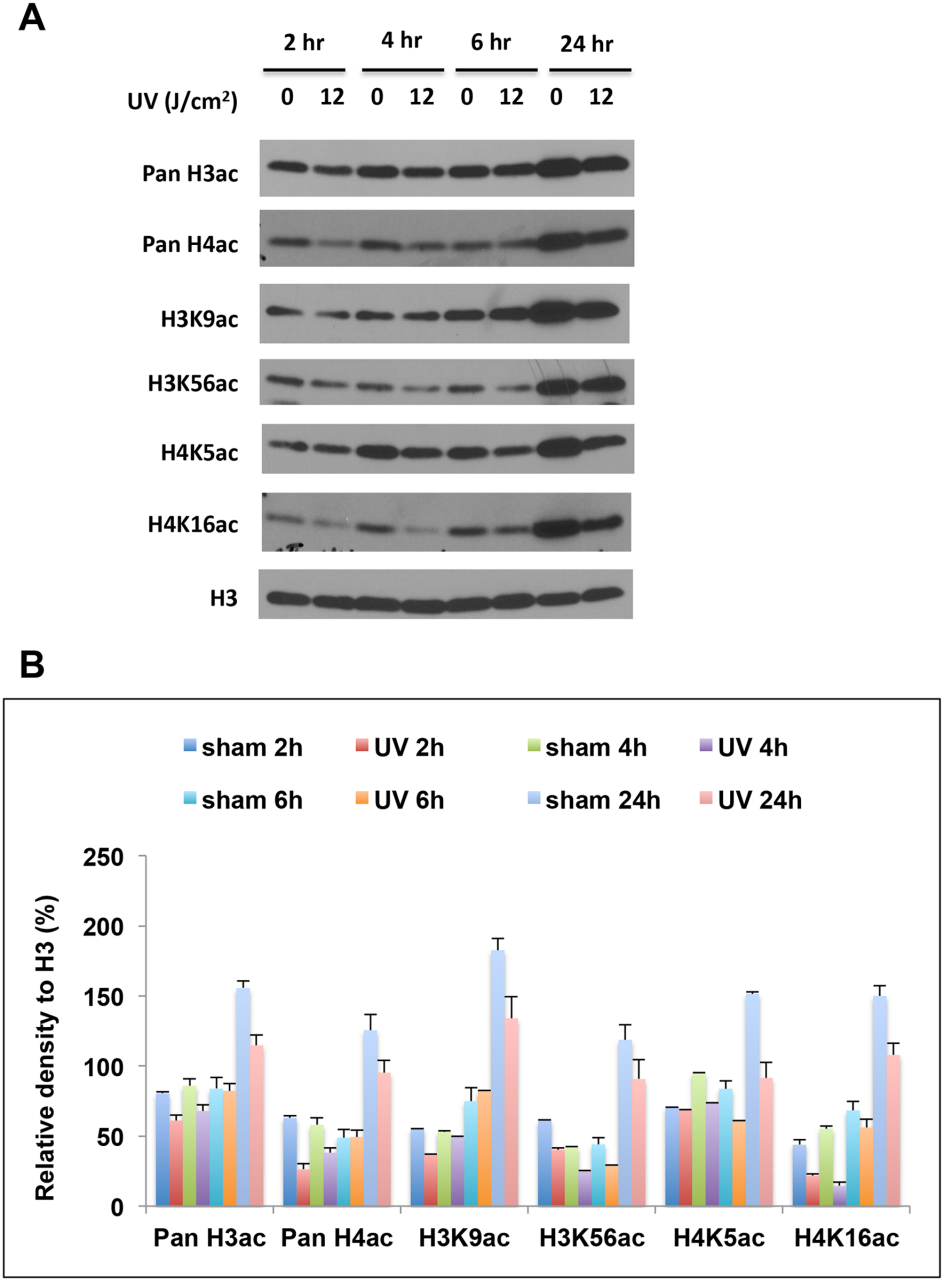
Time course of histone acetylation changes induced by ssUVR. HaCaT cells were exposed to a single ssUVR at 12 J/cm^2^, and cells were harvested at 2-, 4-, 6- and 24-hours after irradiation. (A) Representative western blot. (B) The relative intensity of the bands was presented as the ratio of each band versus its corresponding H3. The results from replicate experiments were plotted as the mean ± SD (n = 3).

To investigate how early these changes occur, we exposed cells to a single 12 J/cm^2^ ssUVR, and collected cell lysate at 2, 4, 6 and 24 hours after irradiation. The marks that displayed a clear dose-dependent reduction in response to ssUVR were further analyzed. As shown in Fig 4, the levels of H3K9ac were reduced initially at 2-hour post radiation. The reduction became less obvious at 4- or 6- hour post radiation, but appeared again at 24-hour post radiation, suggesting a biphasic response to ssUVR. In contrast, the reduction in levels of H4K5ac was not apparent until 4 hours and remained for at least 24 hours post irradiation. H4K16 and H3K56 acetylation decreased at all tested time points (Fig 4). Previous studies have shown that H4K16ac exhibited a biphasic response at DNA damage site, and that both H4K16ac and H3K56ac were the targets of histone deacetylases HDAC1 and HDAC2 [21]. Although our results also showed similarity in the response of these two marks, no biphasic response was observed in our study.

### The persistence of changed acetylation marks

**Fig 5:**
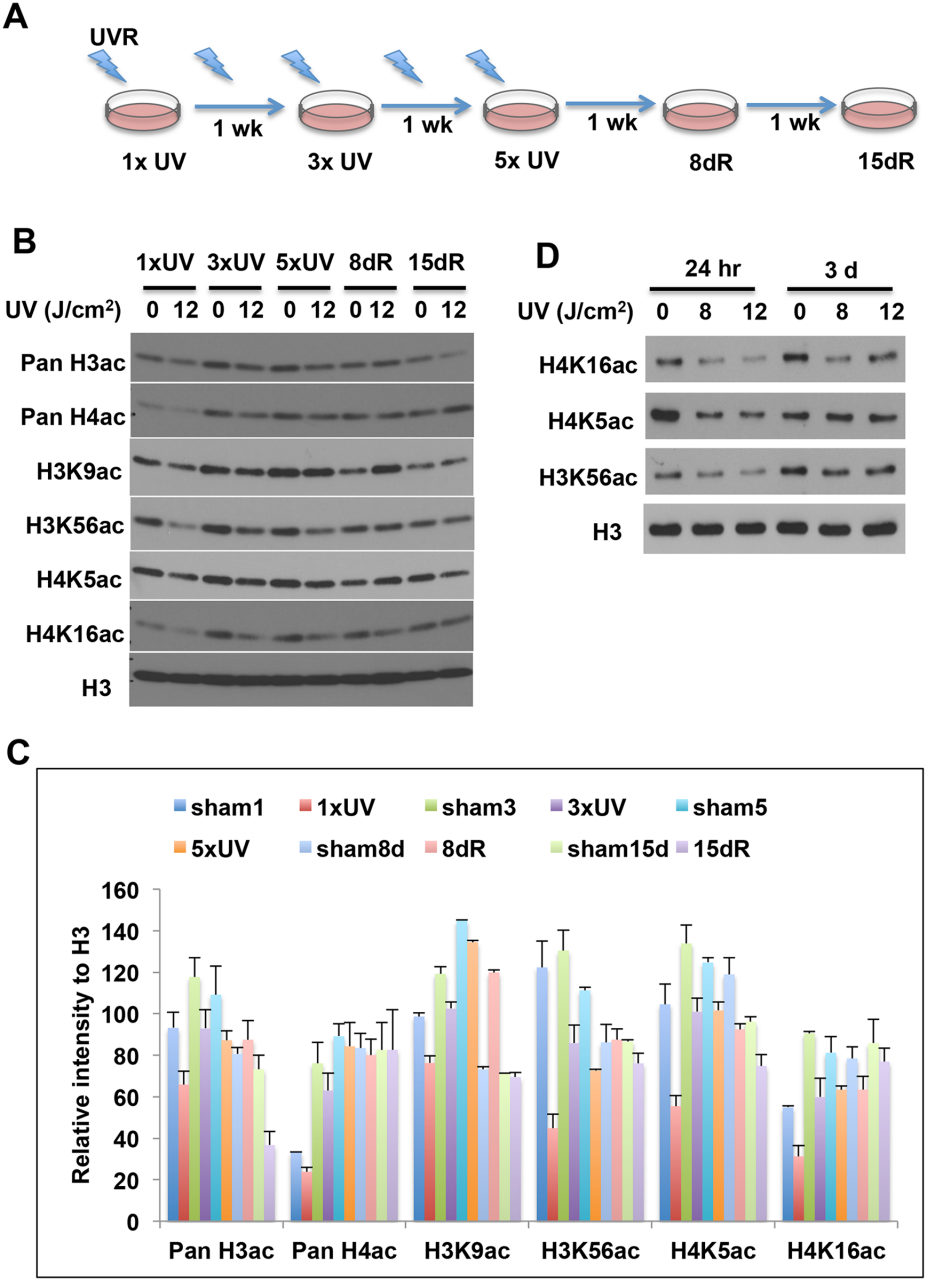
The persistence of histone acetylation following ssUVR exposure. (**A**) Schematic diagram showing the ssUV radiation and recovery of HaCaT cells. (**B**) HaCaT cells were exposed to 5 repetitive ssUVR at 12 J/cm^2^ (once every three days), and then recovered for 15 days following the last irradiation. Cells were harvested at the indicated time intervals. (C) The relative intensity of the bands was presented as the ratio of each band versus its corresponding H3. The results from replicate experiments were plotted as the mean ± SD (n = 3). (**D**) NHEK cells were exposed to a single ssUVR at 8 or 12 J/m^2^, and cells were harvested at 24 hr or 3 days after irradiation. The levels of histone H3 and H4 acetylation were analyzed by western blot using specific antibodies. The relative intensity of the band was measured and is shown below each band. An antibody against histone H3 was used to access the loading of samples in each lane.

Histone PTMs are dynamically regulated to modulate the cellular response to environmental change. Once the stimulus is removed, the altered PTMs can follow two distinct pathways: revert to the original state and re-establish the plasticity and readiness for a new challenge; or retain the change and convert it into a state that is stable and heritable through cell division (epigenetic alteration). To test whether the PTMs changes induced by ssUVR are reversible or persistent after UV radiation, we treated HaCaT cells with 5 repetitive doses (once every three days) of ssUVR at 12 J/cm^2^, and then allowed cells to recover for 8 or 15 days (Fig 5A for experimental design). Cell lysates were collected at 24 hours after 1st, 3rd or 5th repetitive ssUVR, and 8 or 15 days after the last irradiation. As shown in Fig 5B and 5C, the levels of H3K9ac were reduced 24-hours after 1^st^ and 3^rd^ irradiation but recovered by the 5^th^ irradiation. H3K56ac and H4K5ac levels were highly sensitive to UVR exposure during the treatment period (one week). However, the reduction disappeared during the recovery periods. H4K16ac remained low as long as 8 days after the last irradiation. Similar phenomena were observed in NHEK cells. The levels of H4K16ac and H3K56ac remained low at 3 days after a single dose of 8 or 12 J/cm^2^ ssUVR, while H4K5ac levels returned to control levels at same time point (Fig 5D). These results suggested that the persistence of the changed marks varies among different acetylation sites.

### ssUV radiation modulates the activities of histone acetyltransferases and deacetylases

The level of histone acetylation is tightly controlled by the enzyme activities of histone acetyltransferases (HATs) and deacetylases (HDACs). While HATs catalyze the transfer of the acetyl group from acetyl CoA to histone lysine residues, HDACs remove acetyl groups from histones. Since ssUVR exposed cells displayed a hypoacetylation across multiple sites of histone H3 and H4, we reasoned that ssUVR might alter the activities of nuclear HDACs or HATs.

**Fig 6:**
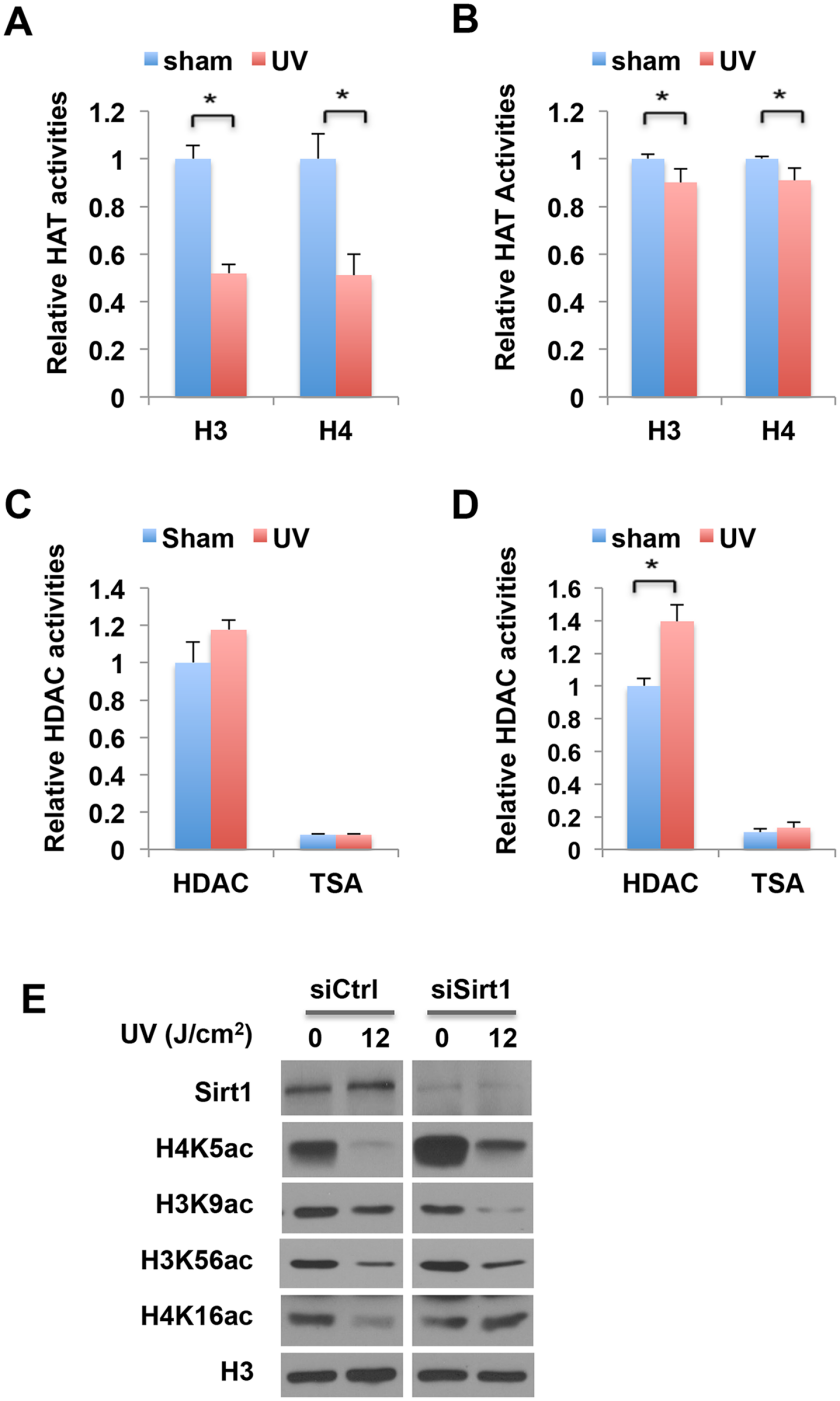
ssUVR modulation of nuclear HAT and HDAC activities in human keratinocytes. (**A, B**) The effect of ssUVR on HAT activity in HaCaT cells (**A**) and NHEK cells (**B**). Nuclear fractions from sham and ssUV radiated cells were isolated and histone H3 and H4 specific HAT activities were measured. Data represent the average of three (HaCaT, n = 6) or two (NHEK, n = 4) independent experiments with duplicates. * P<0.05. (**C, D**) The effect of ssUVR on HDAC activity in HaCaT cells (C) and NHEK cells (D). Nuclear fractions from sham and ssUV radiated cells were isolated and analyzed for HDAC activities. TSA (5 μM) was added to the assay to inhibit class I/II HDAC activities. Data represent the average of two independent experiments with duplicates (n = 4). * P<0.05. (**E**) The effect of Sirt1 knockdown on ssUVR induced histone hypoacetylation. HaCaT cells were transfected with RNAi oligos against human SIRT1 or control RNAi. 72 hours after transfection, cells were exposed to a single ssUVR at 12 J/cm^2^. Cells were collected at 24 hours after irradiation, and analyzed for Sirt1 and histone acetylation. Antibody against histone H3 was used to access the equal loading of samples in each lane.

To identify which process is more likely the target of ssUVR, we measured the enzyme activity in nuclear extracts from ssUV-radiated cells and compared them with sham treated cells. Using histone H3 and H4 peptides as the substrates, we identified that HAT activities were reduced about 30–40% in ssUVR treated HaCaT cells, which is consistent with our observation of hypoacetylation in both histone H3 and H4 (Fig 6A). The ssUVR-induced decrease of HAT activity was also seen in NHEK cells (Fig 6B). The activities of total HDACs (without any inhibitor) were analyzed, while no significant change was observed in HaCaT cells exposed to ssUVR (Fig 6C), a significant increase of HDAC activity was seen in ssUVR treated NHEK cells (Fig 6D). Addition of TSA, a class I/II HDAC inhibitor, abolished more than 90% HDAC activities, suggesting the majority of nuclear HDAC activity measured is from class I/II HDACs. These results suggested that changes in both HATs and HDACs activity might contribute to reduced histone acetylation by ssUVR.

The activity of class III HDACs was undetectable using HDAC activity assay, therefore, we took another approach to determine whether SIRT1, the most abundant class III HDAC in the nucleus, could mediate ssUVR induced histone hypoacetylation. SIRT1 has a site preference for H4K16 and H3K9, and depletion of SIRT1 in human U2OS cells leading to hyperacetylation of both H4K16ac and H3K9ac has been reported [22]. To examine whether SIRT1 is required for ssUVR-induced H4K16 deacetylation, SIRT1 was depleted in HaCaT cells using small interfering RNA (RNAi). As shown in Fig 6E, knockdown of SIRT1 in HaCat cells did not change the basal level of H4K16ac, however, ssUVR induced hypoacetylation of H4K16 was completely abolished in SIRT1 knockdown cells, indicating SIRT1 involvement in ssUVR-induced H4K16 deacetylation. Depletion of SIRT1 resulted in a striking increase of H4K5ac basal level in HaCaT cells, revealing that H4K5 is one of major targets of SIRT1 (Fig 6E). Interestingly, SIRT1 knockdown was unable to rescue the ssUVR-induced decrease of H4K5ac, suggesting that reduced H4K5ac is likely mediated by impaired histone acetylation, which is consistent with the significantly reduced HAT activity in ssUV irradiated cells (Fig 6E).

Taken together, our results demonstrated that ssUV radiation primarily impacted both HAT and HDAC activities, leading to decreased histone acetylation.

## Discussion

Exposure of human skin to UVR results in DNA damage and ROS production. Histone modifications and chromatin structure change are known to occur dynamically in response to these stimuli and play an important role in coordinating the cellular damage response and maintaining genome integrity. The present study investigated the global changes in histone PTMs induced by ssUVR. Decreases in H3K9ac, H3K56ac, H4K5ac, and H4K16ac were found in human keratinocytes exposed to ssUVR. These acetylation changes were associated with ssUVR exposure in a dose-dependent and time-specific manner. Interestingly, H4K16ac, a mark that is crucial for higher order chromatin structure [23] exhibited a persistent reduction that was transmitted through multiple cell divisions. Moreover, the enzymatic activities of HATs and HDACs were affected by ssUVR. The reduced activities of HAT combined with the enhanced HDAC activities likely contributed to the ssUVR-induced hypoacetylation.

Unlike other histone modifications, acetylation alters the charge of lysine residues, allowing chromatin remodeling toward a more open conformation, facilitating the interaction among DNA, histone and other high molecular protein complex [8]. Given its nature, dynamic histone acetylation has been found in all stages of DDR, including damage sensing and signaling, DNA repair, the pause and resumption of replication and transcription, as well as restoration of chromatin structure [5, 24]. Increases and decreases of histone acetylation have both been reported in mammalian cells exposed to DNA damaging agents. In our study, reduced acetylation can be seen at 2 hours after irradiation, suggesting that it may play a role in the early response to DNA damage. These results were consistent with previous studies in human osteosarcoma (U2OS) cells in which H3K9ac, H3K56ac and H4K16ac were reduced shortly after the induction of DNA damage [20, 21, 25]. Interestingly, H4K16ac levels in HaCaT cells were reduced at all time points, even at 8–15 days after ssUVR, while those in U2OS cells were only transiently reduced and the levels increased again 2 hours later [21]. A similar result on the relatively long-lasting loss of H3K9ac, H3K56ac, H4K5ac and H4K16ac after DNA damage has been reported in a recent study using human lymphoblastoid cells [26]. However, several studies have reported increased H3K56ac and H4K16ac following DNA damage, which appear contradictory to our results [18, 27, 28]. It is possible that histone acetylation is highly dynamic process and varies with cell type, source of damage, duration of exposure, as well as DNA repair pathways involved. Another variable that may influence the experimental result is the analysis method of chromatin modification itself. Others have reported alteration of PTMs in damage sites via chromatin immunoprecipitation, while we focused on global changes. In addition, UVA and UVB have very different effects on DNA, and is worth noting that ssUVR used in our study was 5% UVB and 95% UVA, which may be associated with more oxidative DNA lesions than UVB alone.

The ultimate impact of histone hypoacetylation in the cellular response to ssUVR is still not entirely clear although our study and others have shed some light. First, the drastic reduction of acetylation across several marks suggests a possible role involving global chromatin closure. While increased local acetylation provides an open conformation for the repair complex to access the damaged site, a global hypoacetylation may be required to prevent DNA replication and transcription complexes arising from damaged DNA. Indeed, it has been reported that both DNA and RNA synthesis were markedly reduced after cells were exposed to UV radiation [29]. Secondly, reduced acetylation may be actively involved in all steps of DDR, from sensing the damage to successful DNA repair. For example, it has been reported that a period of 24–48 hours are necessary for removing most CPDs induced by UVR [30]. The long-lasting reduction of acetylation observed in our study and others is in line with the repair kinetics of the cells [26, 31]. In one report, DSB-induced local H4K16 deacetylation was required for 53BP1 foci formation and function NHEJ repair [29]. Using time-lapse confocal microscopy, two studies reported local histone hypoacetylation shortly after microirradiation by UV lase, which is crucial for recruiting Oct4 [25] and BMI1 [32] protein to UV-damaged region. Deacetylation of H3K56ac and H4K16ac were critical for successful DNA repair, as cells with HDAC1 and HDAC2 knockdown were unable to repair the DNA damage. Moreover, histone hypoacetylation was most obvious at 24 hours after radiation, suggesting a possible role in chromatin restoration and cell cycle reentry upon the end of DDR. This is supported by previous findings that H4K16ac and H3K56ac are required for chromatin compaction and checkpoint recovery, respectively. Lastly, ssUVR-induced hypoacetylation may alter gene expression profile and control cell death and survival. Recent studies revealed H4K16ac as a critical player in cell death and autophagy [33–36]. Further profiling gene expression changes in these cells will certainly help us understanding the functional impact of histone hypoacetylation in cellular response to ssUV radiation.

How does UV radiation modulate global histone acetylation? Our data suggest that the activities of HATs and HDACs were both affected. Which one is more prominent depends on the specific lysine acetylation site. For example, deacetylation of H4K16ac is more pronounced, as knockdown of SIRT1 almost diminished ssUVR-induced reduction (Fig 6E), while impaired acetylation is a key mediator of ssUVR-induced reduction of H4K5ac. Interestingly, SIRT1 is crucial for maintaining the steady state levels of H4K5ac, however, it was not required for deacetylation of this site induced by ssUVR. It is possible that cells may utilize separate systems to maintain basal levels and to mediate stress response. Other factors may also influence the impact of ssUVR on HATs or HDACs activities. Our results showed that, while HAT activity was significantly inhibited by ssUVR in HaCaT cells, enhanced HDAC activity was more pronounced in NHEKs. It is worth noting that HaCaT carries p53 mutations in both allele and therefore does not have functional p53 protein. It is well known that some HATs and HDACs modulate p53 function via lysine acetylation and deacetylaiton. However, whether p53 affects HATs or HDACs activities remains largely unknown. Given the fundamental role of p53 in DNA damage response, cell cycle checkpoint, and transcription regulation, it would be interesting to study the interplay between p53 and histone modification enzymes.

It is known that histone acetylation is dynamically altered during the cell cycle. The levels of H3K56ac and H4K16ac are normally high in late G1 to S-phase but low during mitosis, while H4K5ac is more abundant in S phase [37]. Given the fact that UV induces G2/M arrest in HaCaT cells, we cannot exclude the possibility that reduced histone acetylation might reflect an altered cell cycle profile [38]. However, several findings suggested that ssUVR-induced hypoacetylation is not a cell cycle dependent event. First, reduced acetylation can be seen at 2–4 hr after irradiation (Fig 4), when there is no apparent activity related to cell cycle. Secondly, H4K16ac remained low at 8–15 days post irradiation when cells have undergone multiple cell divisions (Fig 5). Thirdly, H4K5ac and H4K16ac, which showed a similar pattern during cell cycle progression [37], exhibited a distinct pattern in SIRT1 knockdown cells (Fig 6E), further supported that these changes were independent of cell cycle. Lastly, studies in U2OS cells have demonstrated that DNA damage reduced H3K9ac and H3K56ac is not associated with cell cycle changes [20]. Taken together, these results suggested that altered cells cycle profile by ssUVR is unlikely to be plausible explanation of the observed histone hypoacetylation in our study.

In summary, this is the first report indicating that solar UVR can induce global hypoacetylation in multiple sites on Histones 3 and 4. A more detailed analysis of genome-wide and gene-specific changes in these marks will significantly add to our understanding of UV induced skin damage and carcinogenesis. Our results also indicated that both HATs and HDACs activities were affected by ssUVR. Given the therapeutic potential of HDAC inhibitors, further study of specific enzymes that mediate ssUVR induced epigenetic alteration will provide more insights into future epigenetic therapy.
